# Lock, Stock and Two Different Barrels: Comparing the Genetic Composition of Morphotypes of the Indo-Pacific Sponge *Xestospongia testudinaria*

**DOI:** 10.1371/journal.pone.0074396

**Published:** 2013-09-12

**Authors:** Thomas Swierts, Katja T. C. A. Peijnenburg, Christiaan de Leeuw, Daniel F. R. Cleary, Christine Hörnlein, Edwin Setiawan, Gert Wörheide, Dirk Erpenbeck, Nicole J. de Voogd

**Affiliations:** 1 Marine Zoology, Naturalis Biodiversity Center, Leiden, The Netherlands; 2 Institute for Biodiversity and Ecosystem Dynamics, University of Amsterdam, Amsterdam, The Netherlands; 3 Departamento de Biologia, CESAM, Centro de Estudos do Ambiente e do Mar, Universidade de Aveiro, Aveiro, Portugal; 4 Yerseke Marine Microbiology, Royal Netherlands Institute for Sea Research, Yerseke, The Netherlands; 5 Department of Earth and Environmental Sciences, Palaeontology & Geobiology, Ludwig- Maximilians-Universität München, München, Germany; 6 GeoBio-Center^LMU^, Ludwig-Maximilians-Universität München, München, Germany; 7 Bayerische Staatssammlung für Paläontologie und Geologie, München, Germany; King Abdullah University of Science and Technology, Saudi Arabia

## Abstract

The giant barrel sponge *Xestospongiatestudinaria* is an ecologically important species that is widely distributed across the Indo-Pacific. Little is known, however, about the precise biogeographic distribution and the amount of morphological and genetic variation in this species. Here we provide the first detailed, fine-scaled (<200 km^2^) study of the morphological and genetic composition of *X* *. testudinaria* around Lembeh Island, Indonesia. Two mitochondrial (CO1 and ATP6 genes) and one nuclear (ATP synthase β intron) DNA markers were used to assess genetic variation. We identified four distinct morphotypes of *X* *. testudinaria* around Lembeh Island. These morphotypes were genetically differentiated with both mitochondrial and nuclear markers. Our results indicate that giant barrel sponges around Lembeh Island, which were all morphologically identified as *X* *. testudinaria* , consist of at least two different lineages that appear to be reproductively isolated. The first lineage is represented by individuals with a digitate surface area, CO1 haplotype C5, and is most abundant around the harbor area of Bitung city. The second lineage is represented by individuals with a predominantly smooth surface area, CO1 haplotype C1 and can be found all around Lembeh Island, though to a lesser extent around the harbor of Bitung city. Our findings of two additional unique genetic lineages suggests the presence of an even broader species complex possibly containing more than two reproductively isolated species. The existence of *X* *. testudinaria* as a species complex is a surprising result given the size, abundance and conspicuousness of the sponge.

## Introduction

Marine sponges are diverse and structurally important components of coral reefs [1,2,3]. They provide a substrate for numerous organisms, are involved in marine nutrient dynamics, and are a key source of pharmaceutical compounds [4,5,6,7]. Many aspects of their biology, biogeography and genetic structuring across space and time, however, remain unknown [8,9,10]. This is in large part due to a paucity of variable single-copy markers with sufficient resolution to differentiate between sponge taxa and populations compared to other marine organismal groups [8,10].

The broad distribution of many marine organisms, including sponges, has been attributed to the lumping of morphologically similar but often evolutionarily distinct lineages into single species [11,12]. Molecular studies have indicated the presence of multiple genetically differentiated lineages within morphologically identical samples, i.e. cryptic species [13,14,15,16]. Molecular studies of sponges from the Indo-Pacific are scarce [17,18,19], particularly in the Coral ,Triangle. The Coral ,Triangle is known to be region of highest marine biodiversity and consequently represents an important region for conservation and economic management [20,21].

**Figure 1:**
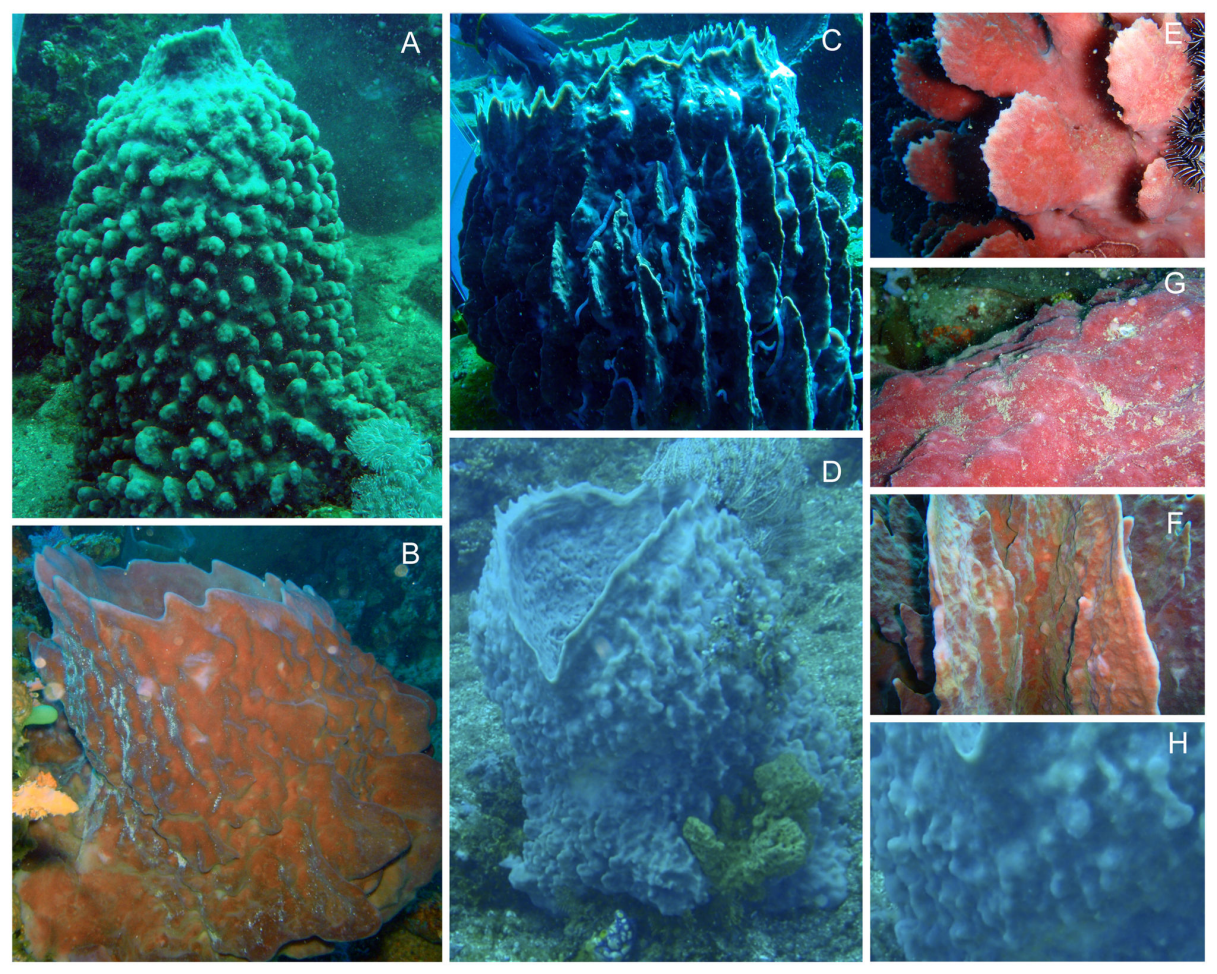
Pictures of identified morphotypes of *Xestospongiatestudinaria* around Lembeh Island. a: Digitate; b: Smooth; c: Lamellate; d: Intermediate; e: Digitate close-up; f: Smooth close-up; g: Lamellate close-up; h: Intermediate close-up.

Giant barrel sponges *Xestospongiatestudinaria* (Lamarck, 1813) and *Xestospongiabergquistia* (Fromont, 1991) in the Indo-Pacific and *Xestospongiamuta* (Schmidt, 1870) in the Caribbean, are among the largest known sponges (Demospongiae; Haplosclerida), measuring up to 2.4 meters in height and width. These iconic animals have a large, erect, barrel-shaped appearance and individuals vary in size, shape (Figure 1) and biochemical composition. The external morphology of giant barrel sponges can vary from smooth to highly digitate or lamellate surfaces [22]. They have very long life spans (possibly in excess of 2000 years); *X* *. muta* has been called the 'redwood of the reef' due to its size, longevity and ecological importance [23]. So far, *X* *. bergquistia* is only known from the northern Great Barrier Reef where it is sympatric with *X* *. testudinaria* . In contrast, the known biogeographical distribution of *X* *. testudinaria* extends from the Red Sea and East Africa to the Great Barrier Reef in Australia and Tonga [2,24]. Its almost ubiquitous presence in coral reefs makes it a good model species to study detailed spatial patterns of morphological and genetic variation in sponges.

The sponges *X* *. testudinaria* and *X* *. muta* share many characteristics. Similar morphologies (smooth, digitate, lamellate) have been described for both species [22,25,26] and their mitochondrial Cytochrome Oxidase 1 (CO1) sequences revealed little divergence between both taxa (99-100% identical). Furthermore, both species displayed similar bacterial community compositions [27]. The above mentioned resemblances prevail despite the fact that these taxa must have been separated for at least three million years, since the closing of the Isthmus of Panama [28]. Hence, it has been proposed that the most significant difference between *X* *. testudinaria* and *X* *. muta* is the geographic region in which they occur [27].

Strong spatial genetic structuring based on CO1 sequence data was found for samples of *X* *. muta* in Caribbean reefs. This structuring was suggested to be related to patterns of ocean currents [26]. While the Caribbean *X* *. muta* is currently among the most intensively studied sponge species in the world, comparative studies of the Indo-Pacific *X* *. testudinaria* are lacking. This hampers our understanding of the evolutionary history of giant barrel sponges and specifically, the relationship between *X* *. testudinaria* and *X* *. muta* . Moreover, the link between external morphotype and genealogy remains unclear, as suggested elsewhere [26,29].

Here, we provide the first detailed, fine-scaled (<200 km^2^) study of the morphological and genetic diversity of *X* *. testudinaria* . Our study took place in the reefs surrounding Lembeh Island (northeast Sulawesi, Indonesia). Our main goal was to test for differences in genetic composition among the morphotypes of *X* *. testudinaria* . We were particularly interested to assess whether this conspicuous sponge taxon represents a species complex, as opposed to a widespread single species. To achieve our goal, individuals of *X* *. testudinaria* were sampled around Lembeh Island, identified to morphotype and sequenced at the I3-M11 partition of the mitochondrial CO1 gene [26,30]. In addition to this, we sequenced the mitochondrial adenosine triphosphate synthase subunit 6 (ATP6) gene [31] and the nuclear adenosine triphosphate synthase β (ATPSβ) intron for a subset of individuals [18,19]. Nuclear genetic markers evolve independently from mitochondrial markers. Hence, congruent patterns across marker types, including morphology, mitochondrial, and nuclear genetic markers, would corroborate the existence of reproductively isolated units, i.e. distinct biological species, e.g. [32,33,34].

## Materials and Methods

### Sampling

Samples of *X* *. testudinaria* were collected by SCUBA diving from 33 different sites around Lembeh Island, off the northeast coast of Sulawesi, Indonesia from January 30^th^ to February 18^th^ 2012 (Table 1). All collections were done during the International Seminar on Conservation of Marine Biodiversity, UNSRAT (University Sam Ratulangi, Manado) as part of the Marine Biodiversity workshop based at the field station of the Research Centre for Oceanography of the Indonesian Institute of Sciences (in Indonesian, Lembaga Ilmu Pengetahuan Indonesia, or LIPI) in Bitung. The workshop was hosted by the Sam Ratulangi University in cooperation with LIPI. We were allowed to collect samples for our research based on a Memorandum of Understanding between LIPI and the Netherlands Centre for Biodiversity Naturalis in Leiden, The Netherlands. LIPI is the governmental authority for science and research in Indonesia. It consists of 47 research centers in the fields ranging from social to natural sciences. LIPI is the authority to collect and export samples. We operated from out of their Research Station in Bitung in cooperation with the Sam Ratulangi University. This university is also authorized to collect by LIPI.

**Table 1:** Specifications of sampled dive sites around Lembeh Island.

**Dive Site Number**	**Dive Site Name**	**Date**	**Coordinates**	**CO1**	**ATP6**	**ATPsβ**
1	Tanjung Nanas I	30-Jan-12	1°27'40.43″N; 125°13'36.41″E	3	3	1
2	SE Sarena Kecil	30-Jan-12	1°27'15.80″N; 125°13'29.53″E	2	2	1
3	E Sarena Besar	31-Jan-12	1°27'34.16″N; 125°14'01.90″E	1	1	1
4	Tanjung Mawali	31-Jan-12	1°26'36.42″N; 125°13'45.98″E	4	4	2
5	Tanjung Nanas II	01-Feb-12	1°27'43.67″N; 125°13'41.63″E	3	3	0
6	Tanjung Kubur	01-Feb-12	1°28'44.69″N; 125°14'59.14″E	1	1	1
7	Pantai Perigi	02-Feb-12	1°28'10.02″N; 125°14'38.80″E	4	1	1
8	Tanjung Nanas I	03-Feb-12	1°27'40.21″N; 125°13'36.41″E	4	1	1
9	Pulau Abadi	03-Feb-12	1°26'00.74″N; 125°12'22.61″E	4	1	0
10	Tanjung Labuhankompeni	04-Feb-12	1°25'55.85″N; 125°11'10.64″E	6	1	1
11	Kelapadua	04-Feb-12	1°26'08.38″N; 125°12'34.09″E	3	1	0
12	Baturiri	06-Feb-12	1°27'34.70″N; 125°14'23.10″E	3	0	0
13	Lobangbatu	06-Feb-12	1°26'02.65″N; 125°12'09.72″E	2	1	0
14	SW Sarena Kecil	07-Feb-12	1°27'19.84″N; 125°13'25.03″E	6	3	2
15	Lobangbatu Besar	07-Feb-12	1°25'49.40″N; 125°11'26.81″E	3	0	0
16	Teluk Rarandam	08-Feb-12	1°27'03.20″N; 125°14'17.52″E	5	1	1
17	Teluk Makawide	09-Feb-12	1°29'05.06″N; 125°14'26.12″E	4	0	0
18	Kelapadua	09-Feb-12	1°26'19.07″N; 125°12'49.00″E	3	0	0
19	Tanjung Kungkungan	10-Feb-12	1°27'58.39″N; 125°14'02.26″E	2	0	0
20	Pulau Abadi	10-Feb-12	1°26'01.03″N; 125°12'22.28″E	2	0	0
21	Tanjung Kuning	11-Feb-12	1°23'10.79″N; 125°10'23.23″E	3	0	0
22	Tanjung Pandea	11-Feb-12	1°23'52.69″N; 125°09'58.93″E	3	3	1
23	N Pulau Dua	13-Feb-12	1°23'28.64″N; 125°12'58.72″E	4	3	1
24	S Pulau Dua	13-Feb-12	1°23'17.02″N; 125°12'43.13″E	6	1	1
25	N Tanjung Pandean	14-Feb-12	1°24'21.71″N; 125°10'04.51″E	3	0	0
26	Desa Pandean	14-Feb-12	1°25'16.07″N; 125°10'52.68″E	4	2	0
27	Teluk Walemetodo	15-Feb-12	1°24'11.34″N; 125°10'20.32″E	1	0	0
28	Tanjung Kelapasatu	15-Feb-12	1°25'38.57″N; 125°11'00.78″E	7	3	2
29	Tanjung Kusukusu	16-Feb-12	1°27'13.75″N; 125°14'12.95″E	5	3	2
30	N Sarena Kecil	16-Feb-12	1°27'26.86″N; 125°13'37.69″E	6	6	2
31	W Sarena Kecil	17-Feb-12	1°27'25.52″N; 125°13'31.19″E	7	5	4
32	Batu Kapal	18-Feb-12	1°32'56.83″N; 125°17'31.85″E	7	2	0
33	Pulau Putus	18-Feb-12	1°31'20.75″N; 125°16'37.27″E	5	2	0
**Total**				**126**	**54**	**25**

Dive sites, sampling dates and coordinates followed by the number of sequences collected of *Xestospongiatestudinaria* per genetic marker. Mitochondrial DNA markers: Cytochrome Oxidase 1 (CO1) and adenosine triphosphate synthase subunit 6 (ATP6), and nuclear DNA marker: nuclear adenosine triphosphate synthase β intron (ATPsβ).

A long, narrow and sheltered channel with a maximum depth of around 30m in the southern part and 70m in the northern part runs between Lembeh Island and the main island of Sulawesi (see Figure 1). This channel, Lembeh Strait, stretches for more than twelve kilometers and has a width of between one and four kilometers. The port of Bitung is located on the main island of Sulawesi, facing the southern part of Lembeh Island. This results in busy shipping traffic in this part of the strait. Lembeh Island lies within the center of maximum marine biodiversity, located in the Indo-Malayan region [20,35].

All sponges were photographed with a digital camera from above and in profile with a unique tag number. A fragment of about 20 grams of each sponge was collected using an apple corer. For each living sponge, we recorded habitat and depth in addition to details of outer morphology and dimensions. After each sampling dive, sponge tissue for DNA extraction was immediately stored in absolute ethanol (98%) in a cool box. After 6-12 hours, the ethanol was changed and samples were stored at -20°C.

Top-view and profile pictures of all sampled giant barrel sponges were independently analyzed by two of us (TS and NJV) and sorted into four different morphotypes (Figure 1a-f) according to the morphotypes described for *X* *. muta* [22,34]. Individuals that were too small to be assigned to a morphotype or for which there was no consensus were classified as ‘Unknown’ (X). The main morphotypes were 'digitate' (D), 'lamellate' (L), 'smooth' (S) and 'intermediate' (I). Digitate sponges had digitate or spiky projections covering their outer body surface (Figure 1a, 1e). Lamellate sponges had closely spaced, pronounced and smooth flanges extending from the base to the apex of their exterior (Figure 1c, 1f). Smooth sponges had a smooth surface area with no surface projections present (Figure 1b, 1g). Intermediate sponges had a rough or bulged surface area in which no distinct pattern was detectable (Figure 1d, 1h).

### Molecular analysis

DNA was extracted from sponge tissue using the DNeasy Blood & Tissue kit (Qiagen) following the instructions of the manufacturer. We sequenced 126 samples from reefs surrounding Lembeh Island for the CO1 mitochondrial gene following [36]. The primers used were C1-J2165 (GAAGTTTATATTTTAATTTTACCDGG) and C1-Npor2760 (TCTAGGTAATCCAGCTAAACC), which amplified a fragment of 544 base pairs (bps). Amplification was performed in a 25µl total reaction volume with: 13.95µl sterile water, 5µl dNTPs (1 mM), 2.5µl 10x buffer, 1.5µl MgCl_2_, 0.4µl forward primer, 0.4µl reverse primer, 0.25µl Taq polymerase and 1µl DNA. PCR profiles consisted of an initial denaturing step (95°C for 5 min), followed by 35 cycles of denaturing (95°C for 30 s), annealing (42°C for 45 s) and extension (68°C for 1.30 min), and a final extension step (72°C for 10 min) executed in a Biorad DNAengine model ptc-200 PCR machine. Following PCR, amplification success was checked on a 1.5% agarose gel. A subset of 54 samples was sequenced for the ATP6 mitochondrial gene following [35], using primers ATP6porF (GTAGTCCAGGATAATTTAGG) and ATP6porR (GTTAATAGACAAAATACATAAGCCTG), which amplified a product of 445 bps. Variation in the mitochondrial genome is typically low for sponges [8,10,17] and the more variable ATP6 gene has been shown to provide valuable additional information [31]. In this subset all CO1 haplotypes were represented by at least one ATP6 sequence. Amplifications were performed in a 20µl total reaction volume containing 9.6µl sterile water, 4µl dNTPs (1 mM), 4µl 5x Phire® Reaction Buffer, 0.3µl forward primer, 0.3µl reverse primer, 0.3µl Phire® Hotstart Taq polymerase DNA (Thermo Scientific, Finnzymes), and 1.5µl DNA. PCR profiles consisted of an initial denaturing step (98°C for 3 min), 35 cycles of denaturing (98°C for 5 s), annealing (38°C for 5 s) and extension (72°C for 20 s), and a final extension step (72°C for 1 min). Sequencing was performed by Macrogen Europe using the PCR primers.

Sequences were checked using CodonCode Aligner version 3.7.1.2 (CodonCode Corporation). primer sequences were trimmed and a final alignment was obtained using CLUSTALW in MEGA 5.05 [37]. Total sequence length was 544 bps for the CO1 alignment and 445 bps for the ATP6 alignment. The best matches from BLAST searches of GenBank for CO1 and ATP6 sequences were with sequences from *X* *. muta* , 99-100% identity (EU716652-EU716655 [30], and 99% identity EU237490 [38], respectively).

To test for congruent patterns at an independent genetic locus, the ATPSβ nuclear intron was amplified for a subset of 25 samples using primers modified from [39]. The ATPSβ intron was chosen because it has proved to be useful and informative in previous studies [18,19]. Detailed methods for this new genetic marker for sponges are described in [18,19]. All sequences are deposited at NCBI Genbank under accession numbers KC424439-KC424444 (CO1), KC424445-KC424447 (ATP6) and KF577733-KF577766 (ATPSβ).

### Data analysis

Mitochondrial CO1 haplotypes were identified and genetic summary statistics were calculated in Arlequin version 3.11 [40]. Statistical parsimony networks displaying evolutionary relationships between haplotypes were obtained with TCS v 1.21 [41]. We combined CO1 haplotypes of *X* *. testudinaria* with data from *X* *. muta* reported in [26] (Genbank accession numbers EU716652-EU716655).

We tested for significant differences in CO1 haplotype composition among morphotypes (n=126) using the adonis function from the vegan library [42] in R (http://www.r-project.org/). The adonis function is an analysis of variance with distance matrices using permutations (also known as a PERMANOVA [43]) that partitions distance matrices among sources of variation; in this case morphotype. Permutational ANOVA’s and adonis analyses are frequently used to test hypotheses related to species composition [44,45,46,47], but they are a general implementation of an ANOVA framework using distances. In the adonis analysis, a distance matrix of pairwise genetic distances from DNA sequences was the response variable with morphotype as independent variable. The number of permutations was set at 1999; all other arguments used the default values set in the function. The distance matrix of pairwise CO1 distances was generated by first importing a fasta file containing sequences of all individuals into R using the read. dna function from the ape library [48]. The sequences were aligned using the muscle function in ape. Additional arguments for the muscle alignment included -gapopen -400.0, -gapextend -0.1, -seqtype dna, -cluster1 neighborjoining, -cluster2 neighborjoining. Finally, a distance matrix of pairwise distances from CO1 sequences was constructed using the dist.dna function in ape with the model argument set to the TN93 model [49].

In order to compare phylogenetic relationships between samples based on mitochondrial and nuclear loci, we constructed maximum likelihood phylogenetic trees in MEGA v 5.05 [37]. We used two final alignments, the first consisted of a combined mitochondrial dataset of CO1 and ATP6 sequences (54 samples, 989 bps) and the second represented 34 sequences of the nuclear intron ATPSβ (258-270 bps in a total alignment of 278 bps). Gaps in the nuclear data were treated as complete deletion. The best-fit DNA substitution model was selected using the Akaike Information Criterion [50], which were the GTR+I model [51] and the HKY+I model [52] for the mtDNA and nuclear DNA (nrDNA) dataset, respectively. Maximum likelihood trees were rooted using the midpoint rooting method as a suitable outgroup was not available [53,54]. Maximum likelihood bootstrap analyses (1000 replicates) were carried out for both datasets.

## Results

### Morphotypes

**Figure 2:**
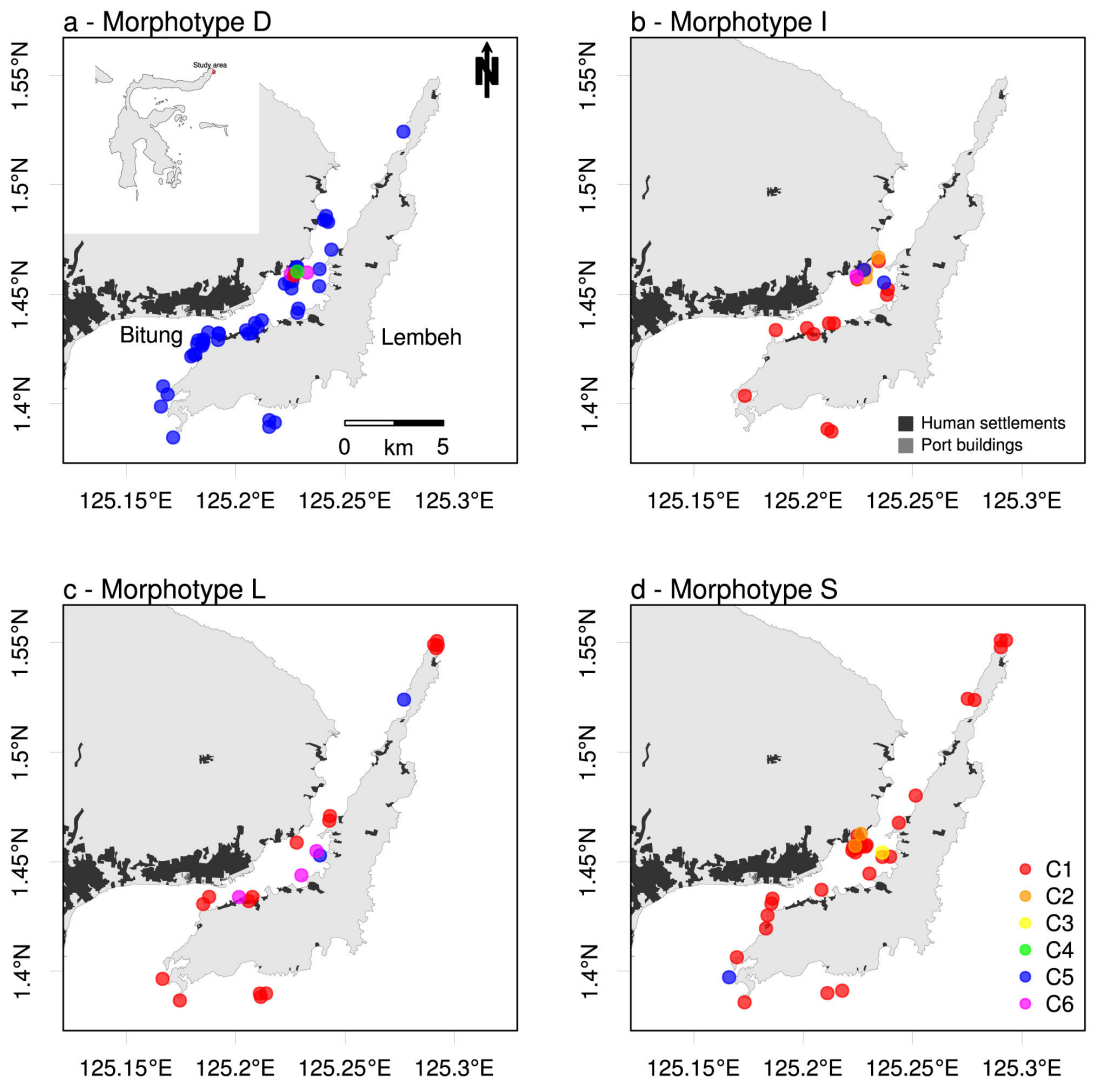
Distribution of mitochondrial haplotypes per morphotype of *Xestospongiatestudinaria* . Spatial distribution of mitochondrial Cytochrome Oxidase 1 (CO1) haplotypes (C1-C6, see also Tables 2 & 3) per morphotype of *Xestospongiatestudinaria* (see Figure 1). C1 (red) and C5 (blue) are present at all sampled sites of Lembeh Island. C2 (orange), C3 (yellow), C4 (green) and C6 (purple) are only present in the sheltered center of Lembeh strait north east of the port of Bitung.

The most abundant morphotype of 126 *X* *. testudinaria* sponges sampled around Lembeh Island displayed the digitate growth form (D, n=47), followed by smooth (S, n=34), intermediate (I, n=21) and lamellate (L, n=19). Five individuals could not be assigned to a morphotype with confidence. The four different morphotypes occurred in close proximity to each other and were present at most of the sampled sites (Figure 2). The digitate morphotype was most abundant in waters adjacent to the harbor buildings and other areas of human settlement of Bitung City (Figure 2A). Morphotypes I, L and S were also present in this area, but in much lower abundances (Figure 2B-D). Morphotype S was most abundant in the center of Lembeh Strait to the north of the harbor area, while morphotype L had a relatively high abundance in the northernmost part of the channel. No relation was found between morphotypes and depth.

### Mitochondrial variation

Genetic composition based on CO1 sequences differed significantly among morphotypes (adonis: F_4, 121_ = 25.42, P < 0.001, R^2^ = 0.457). As can be seen in Figure 2, the major difference in composition was between the digitate morphotype (Figure 2A) and the other morphotypes (Figure 2B-D). In total, 126 CO1 sequences yielded six different haplotypes (named C1-C6) based on a total of four variable sites (Table 2). Two variable sites resulted in non-synonymous substitutions. Haplotypes C1 and C5 were the most abundant haplotypes (n=61 and n=49, respectively) and were found along the entire Lembeh Strait at almost every sampled site (Figure 2). Haplotypes C2, C3, C4 and C6 were far less common (n<10) and were only found in the sheltered center of Lembeh strait to the north of the harbor and city of Bitung (Figure 2). Three mutational steps separate the two most abundant haplotypes; C1 and C5 (see Appendix S1). Haplotypes C2 and C6 represent two smaller groups of sponges (n=6 and n=8, respectively), while only one individual was found representing haplotypes C3 and C4. 

**Table 2:** Nucleotide differences for mitochondrial markers CO1 and ATP6.

**mtDNA**		**CO1**				**ATP6**			**mtDNA combined**
**CO1 (n=126**)	**ATP6 (n=54**)	**11**	**22**	**133**	**463**	**576**	**725**	**785**	**CO1+ATP6 (n=54**)
C1	A1	A	T	A	T	T	T	T	C1A1 (n=18)
C1	A2	.	.	.	.	C	.	C	C1A2 (n=1)
C2	A1	.	.	.	C	.	.	.	C2A1 (n=5)
C3	A1	.	A	.	C	.	.	.	C3A1 (n=1)
C4	A3	.	.	G	C	C	C	.	C4A3 (n=1)
C5	A1	.	A	G	C	.	.	.	C5A1 (n=3)
C5	A2	.	A	G	C	C	.	C	C5A2 (n=19)
C6	A2	G	A	G	C	C	.	C	C6A2 (n=6)

Nucleotide differences in mitochondrial markers Cytochrome Oxidase I (CO1) and adenosine triphosphate synthase subunit 6 (ATP6). Six haplotypes (C1-C6) are found for the CO1 fragment (basepairs 1-544; n=126) with a total of four variable sites. Three haplotypes (A1-A3) are found for the ATP6 fragment (basepairs 545-989; n=54) with a total of three variable sites. Eight different haplotypes are found when the CO1 and ATP6 markers combined (e.g. C1A1, basepairs 1-989; n=54).

Digitate sponges consisted mainly of CO1 haplotype C5 (91.5%), whereas smooth sponges mainly consisted of C1 (88.2%) (Table 3). Mitochondrial diversity estimates for the total dataset (based on CO1 sequences, n=126) were 0.6128 (S.D. =0.0250) (haplotype diversity) and 0.002987 (S.D. =0.001968) (nucleotide diversity). Mitochondrial diversity was much higher for intermediate and lamellate morphotypes (nucleotide diversities of 0.21% and 0.26%, respectively) compared to digitate and smooth morphotypes (nucleotide diversities of 0.046% and 0.071%, respectively).

**Table 3:** Genetic composition and mitochondrial diversity (based on CO1 sequence data, n=126) of *Xestospongiatestudinaria* per morphotype.

Morphotype	N	H	π	C1	C2	C3	C4	C5	C6
Digitate	47	0.1637 (0.0720)	0.000463 (0.000588)	0.021	-	-	0.021	0.915	0.043
Intermediate	19	0.5731 (0.1101)	0.002128 (0.001605)	0.632	0.211	-	-	0.105	0.053
Lamellate	21	0.4095 (0.1205)	0.002574 (0.001834)	0.762	-	-	-	0.095	0.143
Smooth	34	0.2228 (0.0929)	0.000711 (0.000763)	0.882	0.059	0.029	-	0.029	-
Undetermined	5	0.8000 (0.1640)	0.004412 (0.003345)	0.400	-	-	-	0.200	0.400
*Total*	*126*	*0.6128 (0.0250)*	*0.002987 (0.001968)*	*0.484*	*0.048*	*0.008*	*0.008*	*0.389*	*0.064*

Genetic composition and mitochondrial diversity (based on CO1 sequence data, n=126) of *Xestospongiatestudinaria* per morphotype. N = number of samples, H = haplotype diversity, π = nucleotide diversity (standard deviation between brackets). C1-6 refer to six CO1 haplotypes as in Figure 2 and Table 2.

The 54 ATP6 mitochondrial sequences yielded three haplotypes (named A1-A3) identified by a total of three variable sites (Table 2). None of these variable sites resulted in a non-synonymous substitution. The most common haplotypes for ATP6 were A1 (n=26) and A2 (n=27). Haplotype A3 was only found in a single specimen (the same individual in which C4 was found). The concatenated mitochondrial data set resulted in 989 base pairs. All CO1 haplotypes were represented by at least one ATP6 sequence and networks based on both markers were congruent (not shown, but see Table 2). Table 2 also shows that ATP6 contributed additional information, useful for inferring phylogenetic patterns (see combined mitochondrial haplotypes (C1A1-C6A2) for *X* *. testudinaria* ).

### Nuclear variation

**Figure 3:**
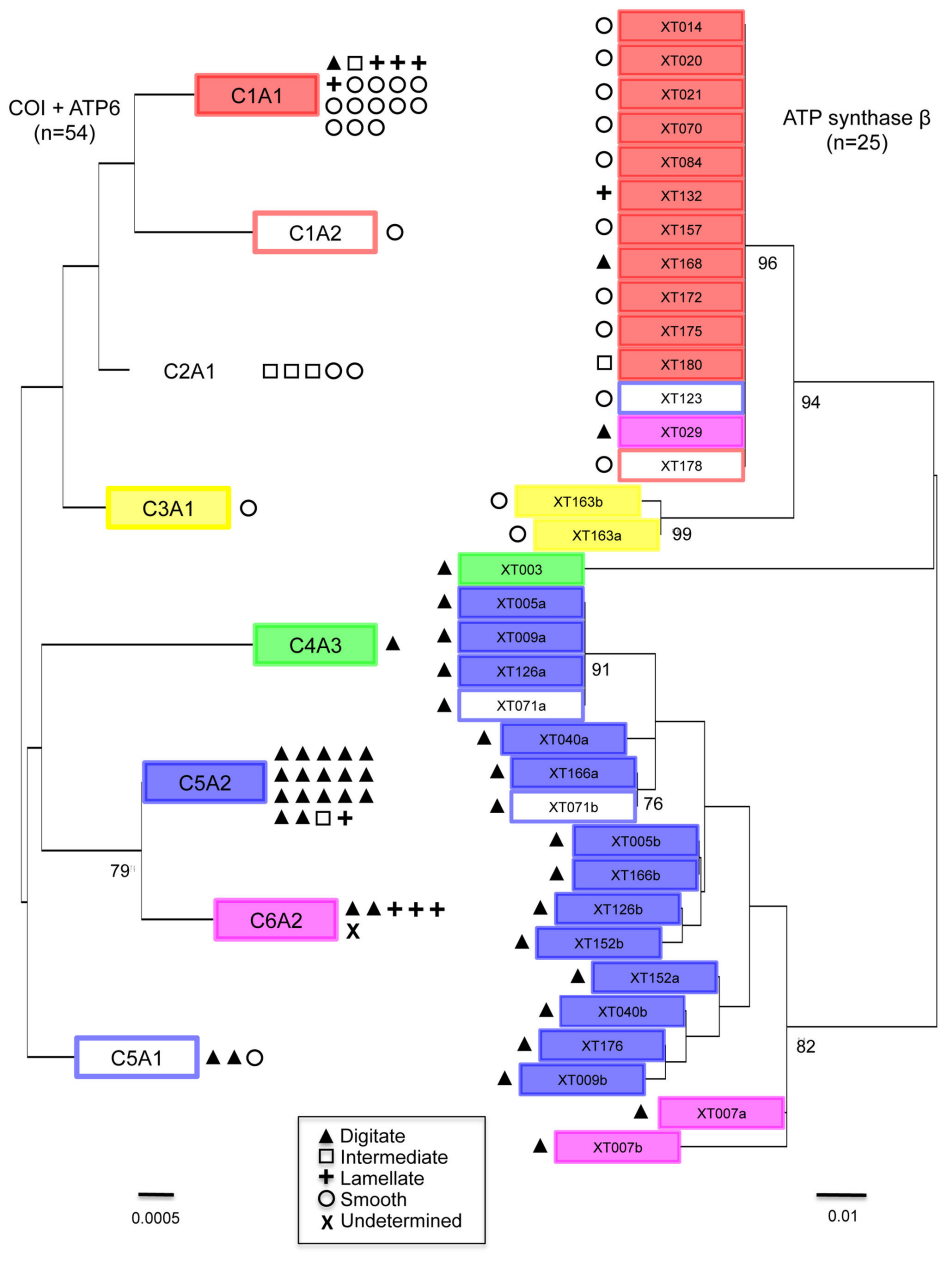
Phylogenetic trees based on mitochondrial and nuclear DNA of *Xestospongiatestudinaria* . Unrooted maximum likelihood phylogenetic trees of the combined mitochondrial DNA (CO1+ATP6, n=54, 989 base pairs, left) and nuclear intron ATPSβ (n=34, 258-270 base pairs, right) sequences of *Xestospongiatestudinaria* . Symbols denote the assigned morphotype of different individuals, with the number of symbols indicating the number of sampled sponges of a given morphotype with that specific DNA sequence (see legend). The colored boxes of the individual sample numbers in the nuclear DNA phylogeny correspond to the same colored boxes of haplotypes in the mitochondrial tree. These colors correspond with the CO1 haplotypes in Figure 2. Haplotypes with an outlined box in a particular color share the CO1 sequence but vary in ATP6 sequence, resulting in a unique combined haplotype (CO1+ATP6). The letters "a" and "b" in the nuclear gene tree represent heterozygote alleles. Bootstrap values are only shown when > 70%. Scale bars depict substitutions per site.

A subset of 25 specimens representing all morphotypes was sequenced for the nuclear ATPSβ intron yielding 16 different alleles with a total of 67 variable sites in a total alignment of 278 bps. Nine individuals were heterozygotes; their allelic sequences are labeled with "a" and "b" (Figure 3). This nuclear DNA (nrDNA) dataset revealed a pattern that is largely consistent with that of the combined mitochondrial DNA (mtDNA) data and morphotype assignment given that individuals sharing mtDNA haplotypes are grouped together in the nrDNA phylogenetic tree as well (Figure 3).

Generally, two major clades are visible in the more resolved nrDNA phylogeny with bootstrap support of 82% and 96%, respectively (Figure 3). The first clade consists of 17 nuclear alleles with mitochondrial haplotypes C5A2 (filled blue box), C5A1 (outlined blue box) and C6A2 (pink box). All samples in this clade are digitate and all but one individual (XT176) are heterozygous. The second major clade consists primarily of mitochondrial haplotype C1A1 (filled red box), however, C1A2, C5A1 and C6A2 are also represented by a single individual each in this clade. All samples in this clade are homozygotes for a shared nuclear allele and the clade consists predominantly of samples with a smooth outer morphology, although other morphotypes (D, L, I) are represented in this clade as well. Two single individuals of unique mitochondrial haplotypes C3A1 (yellow box) and C4A3 (green box) represent unique nuclear genetic lineages as well. All in all, samples with similar mitochondrial haplotypes group together in the nuclear phylogenetic tree as well, with the exception of individuals XT029 and XT123. In this respect, sample XT123 is notable because it is the only sample with mitochondrial haplotype C5A1 that has a smooth morphotype. Sample XT123 grouped together in the nuclear phylogeny with the other smooth samples, whereas the two digitate morphotypes with haplotype C5A1 grouped with the other digitate samples (Figure 3).

## Discussion

Four distinct morphotypes of *Xestospongiatestudinaria* were identified around Lembeh Island, Indonesia. There was, however, a noticeable higher occurrence of sponges with a digitate exterior in waters surrounding the port of Bitung and other human settlements compared to sponges with intermediate, lamellate and smooth morphotypes. Shipping traffic and human activities have been linked to more turbid waters and higher nutrient values [55]. This may favor digitate sponges given that they have higher surface/volume ratios compared to the other morphotypes; the digitate surface projections may also aid in the removal of heavy sediment loads as has been demonstrated for lamellate structures [56]. To better understand the distribution of morphotypes, abiotic measurements such as wave-action, light attenuation and sediment composition need to be taken into account in future studies. The degree and type of morphological variation observed were similar to those described for *X* *. muta* [30,26]. Different functional roles have been proposed to explain the morphological variation, e.g., lamellate structures have been linked to the removal of heavy sediment loads [56]. Nevertheless, no correlation was found between morphology and depth, micro-habitat or geographical locality for *X* *. muta* [22].

We found significant differences in genetic composition among the morphotypes of *X* *. testudinaria* (Figures 2-3), which suggests that these are not mere ecophenotypic varieties, but rather reproductively isolated units. Our total dataset of 126 CO1 sequences showed that haplotype C5 was far more abundant in the digitate morphotype, whereas haplotype C1 was more abundant in intermediate, lamellate and smooth morphotypes (Table 3). ATP6 proved to be a good addition to the CO1 gene to study mitochondrial variation in giant barrel sponges. This yielded more mtDNA variation, but the number of variable sites was still relatively low, hampering phylogenetic support of the clades. This is illustrated by the fact that most clades in the mtDNA tree received relatively low bootstrap support (<70%) (Figure 3). It is well known that sponges (and other non-bilaterian animals) share a comparatively lower degree of mtDNA variation compared to Bilateria [8,10,57]. The nuclear intron sequenced here from *X* *. testudinaria* contained much more genetic variability than the mtDNA fragments, and yielded congruent patterns with the mtDNA phylogeny. Individuals that shared a haplotype for the combined mtDNA (CO1+ATP6) dataset grouped together in the nuclear phylogenetic tree. If extensive genetic exchange is present between the different morphotypes in *X* *. testudinaria* , we would expect a random distribution of both mitochondrial and nuclear genetic types across the phylogenies. This, however, is not the case in *X* *. testudinaria* around Lembeh Island. In the future, we hope to sequence individuals from other areas and to sequence more individuals for the nuclear gene.

**Figure 4:**
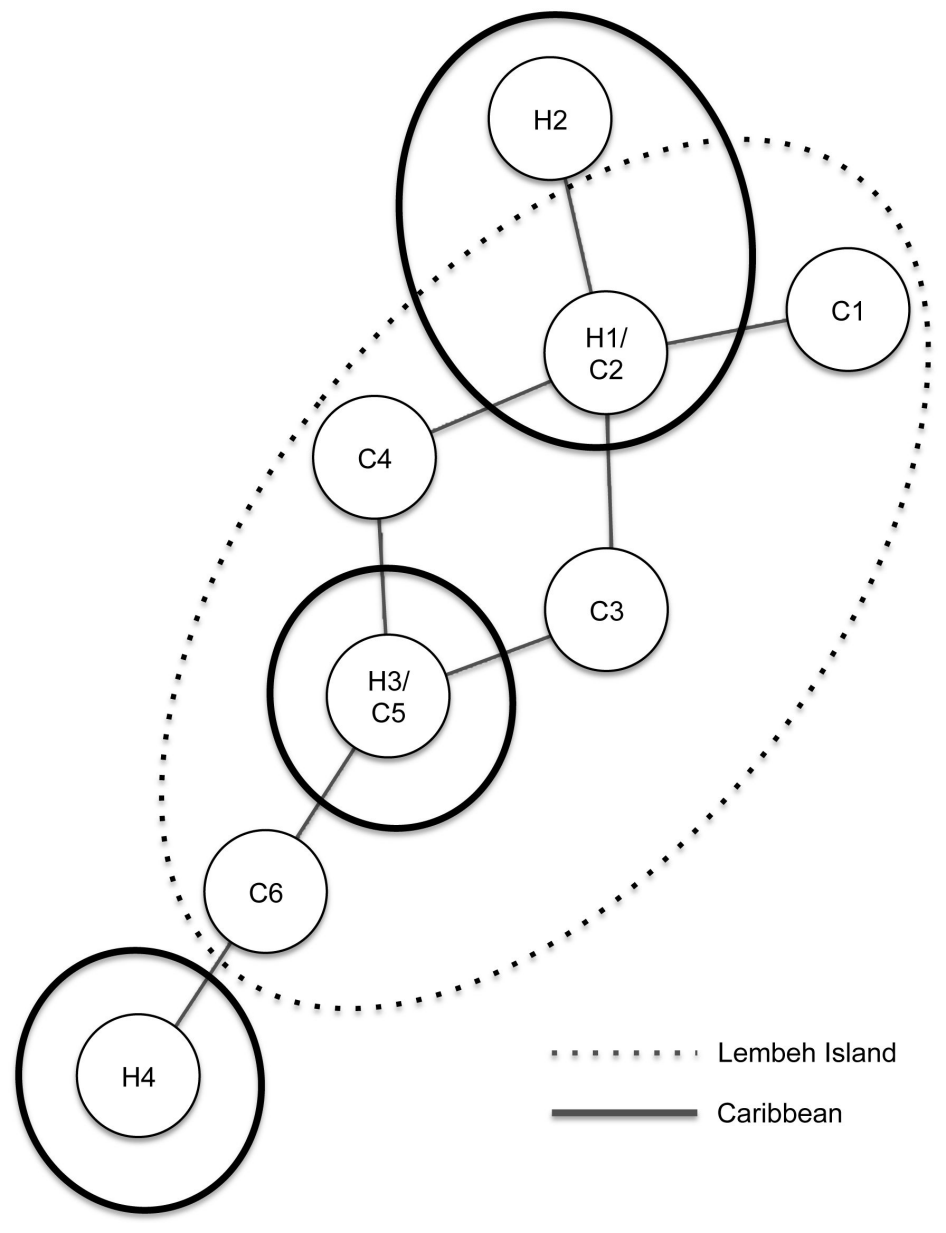
Combined haplotype network of *Xestospongia* *sp.* from the Caribbean and Lembeh Island. Haplotype network of eight Cytochrome Oxidase 1 (CO1) mitochondrial haplotypes of giant barrel sponges *Xestospongiatestudinaria* and *X* *. muta* . Dotted line encircles haplotypes found in our study of 126 *X*. testudinaria around Lembeh Island (C1-6). Solid lines encircle haplotypes found in 116 samples of *X* *. muta* from the Caribbean (H1-4) [26]. Each line connecting the haplotypes represents a single nucleotide substitution.

The mitochondrial and nuclear intron data presented here provide strong support for the existence of a species complex of *X* *. testudinaria* around Lembeh Island. A species complex was previously proposed for the closely related X*. muta* by Kerr and Baker [29] in the Caribbean based on sterol chemistry. Our data show that there are much larger morphological and genetic differences within *X* *. testudinaria* from Lembeh Island than between individuals of *X* *. testudinaria* and *X* *. muta* sharing the same haplotypes and morphotypes (Figure 4). In total, six haplotypes were found for the CO1 mitochondrial fragment in giant barrel sponges around Lembeh Island. López-Legentil & Pawlik [26] only found four haplotypes at this same locus for *X* *. muta* sampled throughout the Caribbean, and thus encompassing a much larger area. Our haplotypes C2 and C5 are identical to haplotypes H1 and H3 respectively, found in *X* *. muta* [26]. The most abundant haplotypes in our dataset, C1 and C5, occurred at all sites around the island. The four other haplotypes, all of which were found in less than ten individuals, only occurred at the sheltered center of Lembeh Strait away from the port and city of Bitung (Figure 2). This undisturbed sheltered area, which has a less oceanic profile compared to sites outside of Lembeh Strait, appears to be a local hotspot of genetic diversity. The exact reason for this particular distribution is unknown [58]. The fact that four of the six haplotypes are only found in a very small portion of the reefs surrounding Lembeh Island illustrates the importance of fine-scaled sampling.

A third giant barrel sponge species, *Xestospongiabergquistia* was described from the Northern Great Barrier Reef. This sponge is sympatric with *X* *. testudinaria* and is very similar in outer morphology and spicule dimensions. The differences between the two species are very subtle with the main difference being that *X* *. testudinaria* has some sponge fiber development around the spicules and therefore has a more compact consistency than *X* *. bergquistia* and it was noted that the live texture is the best field guide to species identification [25]. In the present study, we noticed a large variation in consistency and thus in sponge fiber development, but all of our specimens contained spongin, and were thus assigned to *X* *. testudinaria* . Therefore the possible presence of *X* *. bergquistia* cannot explain the morphological and genetic variation in the giant barrel sponge populations around Lembeh Island and we conclude that at least two distinct, and possibly more, species coexist in the Lembeh Strait. In addition to the dissimilarity in sponge fiber development, *X* *. testudinaria* and *X* *. bergquistia* spawn during different time periods [59]. For Great Barrier Reef sponges, spawning only occurs during a short period between October-November [59,60]. This has been linked to annual changes in seawater temperature [61]. During fieldwork early in 2012, we observed several individuals of *X* *. testudinaria* spawning during the full moon lunar cycle and it has also been observed in August in Ambon [62]. Given that waters around Indonesia show little seasonal variation in temperature [63], it is possible that barrel sponges in these waters spawn during the whole year. The as yet unidentified differences in the timing of spawning events, however, may explain the persistence of a species complex in *X* *. testudinaria* .

The fact that Caribbean and Indo-Pacific giant barrel sponges share CO1 haplotypes is perhaps more surprising than the existence of a species complex around Lembeh Island. After more than three million years of separation [28] the CO1 mitochondrial genes of giant barrel sponges have still not accumulated mutations to differentiate these taxa or alternatively, this may be the result of secondary contact between the two species. Figure 4 shows a combined CO1 haplotype network from this study with results from López-Legentil and Pawlik [26]. This network shows that two individuals from different regions (i.e., Caribbean and Indo-Pacific) can be more closely related than two individuals occurring in sympatry. This strongly suggests that there are in fact different species in the Indo-Pacific and possibly in the Caribbean as well. It is unlikely that barrel sponges from the Indo-Pacific and Caribbean exchange genes nowadays, but this needs to be tested with more variable genetic markers than CO1, such as the nuclear ATPSβ intron that we used.

In summary, giant barrel sponges found around Lembeh Island that are morphologically identified as *X* *. testudinaria* consist of at least two different lineages that probably represent reproductively isolated species. The first lineage has a digitate surface area and mitochondrial CO1 haplotype C5 or C6, and is most abundant around the harbor area of Bitung city. The second lineage is represented by CO1 haplotypes C1 and C2 and usually has a smooth surface area, though other morphotypes have also been found to belong to this lineage. This lineage can be found all around Lembeh Island though to a lesser extent around the harbor of Bitung city. More species may be present in this species complex as some unique genetic lineages for both mitochondrial and nuclear markers were found in the sheltered and relatively undisturbed center of Lembeh Strait. In addition to detailed spatial sampling in the Indo-Pacific, as was done in this study, a wide global-scale sampling of genetic and morphological diversity in giant barrel sponges is necessary to elucidate further biogeographic and evolutionary relationships in this taxon.

## Supporting Information

Appendix S1 Haplotype network based on mitochondrial DNA for *Xestospongiatestudinaria* around Lembeh Island.
(DOC)
