# Supplementary material for: Lock, Stock and Two Different Barrels: Comparing the Genetic Composition of Morphotypes of the Indo-Pacific Sponge Xestospongia testudinaria
Source: PLoS One. 2013 Sep 12;8(9):e74396. doi: 10.1371/journal.pone.0074396 (PMC3771914; doi:10.1371/journal.pone.0074396)
Supplement: Appendix S1 — Haplotype network based on mitochondrial DNA for Xestospongiatestudinaria around Lembeh Island. (DOC) [file pone.0074396.s001.doc]

**Appendix 1. Haplotype network based on mitochondrial DNA for *Xestospongia testudinaria* around Lembeh Island.** Haplotype network of six Cytochrome Oxidase 1 (CO1) mitochondrial haplotypes of 126 *Xestospongia testudinaria* specimens sampled around Lembeh Island. Circle size is proportional to the haplotype frequency. Proportions of colors within each pie chart are representative of the relative number of that haplotype for each morphotype. Each line connecting the haplotypes represents a single nucleotide substitution.
